# Evaluation of the My Diabetes Care Patient Portal Intervention: Protocol for a Pilot Randomized Controlled Trial

**DOI:** 10.2196/25955

**Published:** 2021-05-25

**Authors:** William Martinez, Amber J Hackstadt, Gerald B Hickson, S Trent Rosenbloom, Tom A Elasy

**Affiliations:** 1 Department of Medicine Vanderbilt University Medical Center Nashville, TN United States

**Keywords:** patient portals, self-management, patient activation, diabetes mellitus, type 2, health literacy, health knowledge, attitudes, practice, mobile phone

## Abstract

**Background:**

My Diabetes Care (MDC) is a multi-faceted intervention embedded within an established patient portal, My Health at Vanderbilt. MDC is designed to help patients better understand their diabetes health data and support self-care. MDC uses infographics to visualize and summarize patients’ diabetes health data, incorporates motivational strategies, provides literacy-level appropriate educational resources, and links to a diabetes online patient support community and diabetes news feeds.

**Objective:**

This study aims to evaluate the effects of MDC on patient activation in adult patients with type 2 diabetes mellitus. Moreover, we plan to assess secondary outcomes, including system use and usability, and the effects of MDC on cognitive and behavioral outcomes (eg, self-care and self-efficacy).

**Methods:**

We are conducting a 6-month, 2-arm, parallel-design, pragmatic pilot randomized controlled trial of the effect of MDC on patient activation. Adult patients with type 2 diabetes mellitus are recruited from primary care clinics affiliated with Vanderbilt University Medical Center. Participants are eligible for the study if they are currently being treated with at least one diabetes medication, are able to speak and read in English, are 21 years or older, and have an existing My Health at Vanderbilt account and reliable access to a desktop or laptop computer with internet access. We exclude patients living in long-term care facilities, patients with known cognitive deficits or severe visual impairment, and patients currently participating in any other diabetes-related research study. Participants are randomly assigned to MDC or usual care. We collect self-reported survey data, including the Patient Activation Measure (R) at baseline, 3 months, and 6 months. We will use mixed-effects regression models to estimate potentially time-varying intervention effects while adjusting for the baseline measure of the outcome. The mixed-effects model will use fixed effects for patient-level characteristics and random effects for health care provider variables (eg, primary care physicians).

**Results:**

This study is ongoing. Recruitment was closed in May 2020; 270 patients were randomized. Of those randomized, most (214/267, 80.1%) were non-Hispanic White, and 13.1% (35/267) were non-Hispanic Black, 43.7% (118/270) reported being 65 years or older, and 33.6% (90/268) reported limited health literacy. We obtained at least 95.6% (258/270) completion among participants through the 3-month follow-up assessment.

**Conclusions:**

This randomized controlled trial will be one of the first to evaluate a patient-facing diabetes digital health intervention delivered via a patient portal. By embedding MDC into Epic’s MyChart platform with more than 127 million patient records, our intervention is directly integrated into routine care, highly scalable, and sustainable. Our findings and evolving patient portal functionality will inform the continued development of MDC to best meet users’ needs and a larger trial focused on the impact of MDC on clinical end points.

**Trial Registration:**

ClinicalTrials.gov NCT03947333; https://clinicaltrials.gov/ct2/show/NCT03947333

**International Registered Report Identifier (IRRID):**

DERR1-10.2196/25955

## Introduction

### Background

Diabetes is a leading cause of several highly morbid and costly conditions, including chronic kidney disease, cardiovascular disease, and visual impairment [1]. Attention to diabetes self-management behaviors can help patients avoid or delay many diabetes-related complications; however, consistent engagement in self-care behaviors is challenging for many patients [1,2]. Patient activation (ie, knowledge, skills, and confidence in managing one’s own health) is vital to achieving optimal diabetes self-management and is associated with lower health care costs [3-5].

Patient portals are computerized tools that can connect patients with electronic health data maintained by their health care system. Patient portals can provide an engaging and convenient means for patients to track and visualize health data, obtain education and guidance, and connect patients and doctors [6]. Research has shown that patient portals offer a promising platform to increase patient activation, enhance care, and promote self-management while overcoming the limitations of costly and difficult-to-scale face-to-face interventions [7,8]. We recently applied a user-centered design sprint methodology and key strategies for patient engagement to develop a patient portal intervention called My Diabetes Care (MDC; formerly Diabetes Dashboard) [9].

**Figure 1:**
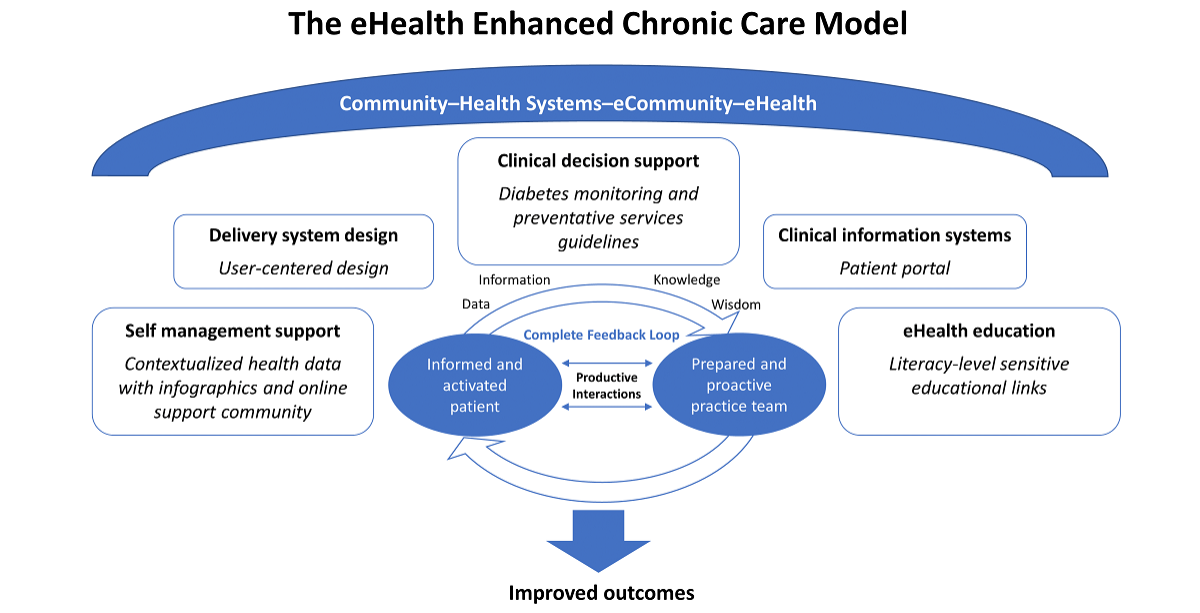
The eHealth Enhanced Chronic Care Model with key aspects of My Diabetes Care shown in italics under the corresponding domain.

MDC is embedded within an established patient portal, My Health at Vanderbilt (MHAV), at Vanderbilt University Medical Center (VUMC) [10]. MDC is a multi-faceted intervention designed to help patients better understand their diabetes health data and support self-management [9]. MDC uses infographics to visualize and summarize patients’ diabetes health data; incorporates motivational strategies (eg, social comparisons); provides literacy-level appropriate educational resources; contains secure messaging capability; and links to a diabetes online patient support community and diabetes news feeds, highlighting new discoveries, medicines, and recipes. MDC was founded on the well-established Chronic Care Model adapted for eHealth—eHealth Enhanced Chronic Care Model (eCCM) [11]. By leveraging elements within the model’s 5 domains (self-management support, delivery system design, decision support, clinical information systems, and eHealth education), MDC has the potential to create more informed and activated patients, leading to improved outcomes (Figure 1).

A 1-month usability study of MDC among 60 patients found that participants, including those with limited health literacy, highly rated MDC’s usability and acceptability [12]. User experience data showed that most participants found that the infographics and links to literacy-level appropriate diabetes health information helped them better understand their diabetes health data. Participant feedback also identified areas for improvement, including adding information about diabetes medications and links to diabetes news feeds, highlighting new discoveries, medicines, and recipes. Consistent with the eCCM, the study also found a significant pre-post increase in patient activation scores among the study participants. However, a larger randomized controlled trial (RCT) is needed to assess MDC’s impact on patient activation more definitively.

### Objectives

This study aims to evaluate the effects of MDC on patient activation in adult patients with type 2 diabetes mellitus (T2DM). In addition, we plan to explore secondary outcomes, including system use and usability, and the effects of MDC on diabetes self-efficacy, knowledge, self-care, medication adherence, distress, and clinical endpoints. The study will serve as a pilot for a larger definitive trial evaluating the effect of MDC on clinical endpoints.

## Methods

### Study Design

To achieve this objective, we are conducting a 6-month, 2-arm, parallel-design, pragmatic pilot RCT of MDC. Participants in both arms are told the purpose of the study is *to determine satisfaction with 2 versions of MHAV among patients with diabetes*. One version is the currently available version of MHAV. The second version of MHAV contains the MDC intervention. Participants in both arms complete the study questionnaires at 3-time points: T_0_=baseline, T_1_=3 months, and T_2_=6 months.

The study protocol is registered with ClinicalTrials.gov (ID NCT03947333) and is being conducted in accordance with the principles outlined in the CONSORT (Consolidated Standards of Reporting Trials) Statement, extension for pragmatic trials [13]. Pragmatic trials are designed to evaluate the real-world effectiveness of interventions in routine practice environments [14]. Unlike a strictly controlled trial, participants in our study are not constrained to receive a controlled *dose* of the intervention [14]. Due to necessity, participants are not blinded to the intervention, and we do not attempt to control participants’ communication or information-seeking behaviors beyond the 2-arm randomization described here. The Vanderbilt University institutional review board approved this study.

### Recruitment and Eligibility

Participants are recruited from 14 VUMC-affiliated adult primary care clinics located throughout Middle Tennessee (4 urban and 10 suburban clinics). An electronic health record (EHR; Epic Systems Corp) stores all clinical data. Patients receive access to their clinical data via an integrated and highly adopted patient portal, MHAV, that is accessible on desktops and via a native mobile app for iOS and Android mobile operating systems.

Participants are eligible for the study if they are a patient at a participating primary care clinic and have T2DM, are currently being treated with at least one diabetes medication, are able to speak and read in English, are 21 years or older, have an existing MHAV account, and have reliable access to a desktop or laptop computer with internet access. We exclude patients living in long-term care facilities, patients with known cognitive deficits, patients with a severe visual impairment, and patients currently participating in another diabetes-related research study.

On a rolling basis, potentially eligible patients are selected from a randomly ordered list of established adult patients with diabetes from participating clinic sites and are sent a recruitment letter describing the study. In addition, we also use *My Research at Vanderbilt* to send the recruitment letter to current patient portal users who elected to allow investigators to contact them about research opportunities via email. Interested patients contact a research assistant to learn more about the study. To enroll, participants complete a web-based study eligibility screener and electronic consent form on the web via REDCap (Research Electronic Data Capture) version 5.0.8. [15].

### Procedures and Randomization

A study coordinator contacts all enrolled participants to review study procedures, answer the remaining questions, and confirm eligibility criteria. The enrolled participants are sent a baseline questionnaire via REDCap. After receiving the completed baseline questionnaire, the study coordinator randomly assigns participants to 1 of 2 groups: (1) intervention or (2) usual care. The randomization sequence was generated by the research team biostatistician using a permuted block randomization scheme stratified by clinic site and participants’ age group (65 years and older vs younger than 65 years) to obtain balance across treatment groups on key variables. The randomized assignment for eligible participants is accessible only to the study coordinator and biostatistician using the REDCap randomization module; the other investigators are blinded. Once a randomization assignment is finalized, participants in both arms receive an email with their treatment assignment and an explanation of how to navigate to features of MHAV specific to their treatment group. Participants are asked to reply to the email affirming that they can access MHAV and/or MDC in accordance with their group assignment. Monthly quality assurance checks are used to ensure that MDC is functioning correctly (eg, displaying data correctly) and to ensure the fidelity of the intervention.

A participant may withdraw from the study at any time by notifying the study team. In addition, participants are withdrawn from the study by the investigators if they do not complete the baseline questionnaire needed for randomization. If a participant is withdrawn from the study for any reason, they are notified, and a reason is provided.

### Intervention and Control

**Figure 2:**
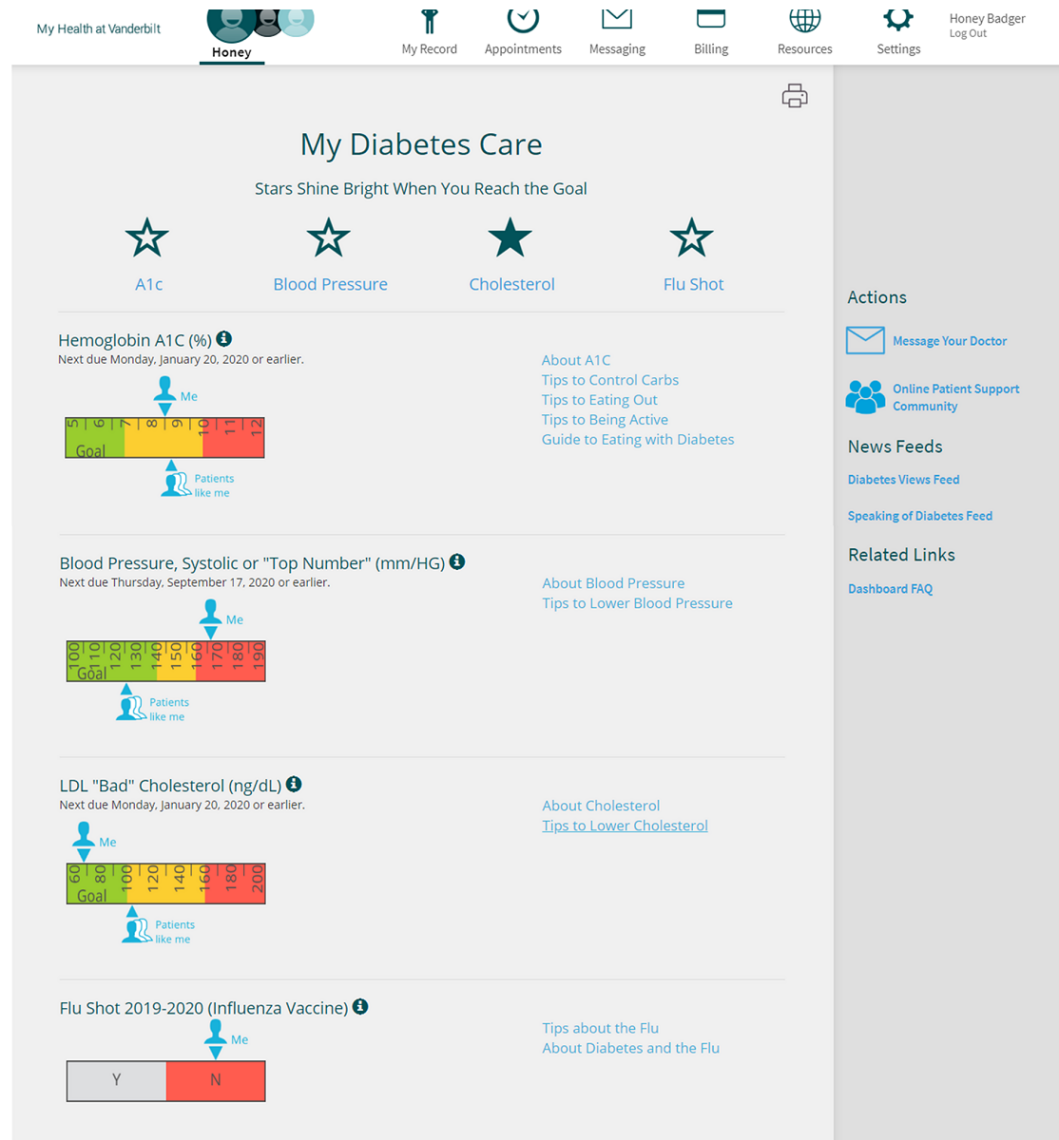
My Diabetes Care screenshot and features. Stars across the top fill in when the patient's glycated hemoglobin, blood pressure, cholesterol, or flu vaccine status are within goal range (ie, a value in the green zone on the infographic for each measure). Info icons provide a brief literacy-level appropriate description of each measure. Infographics display health data relative to a goal (green), caution (yellow), and warning (red) ranges. Patients Like Me indicates the average value of similar patients (ie, Vanderbilt patients with diabetes of the same gender, age group, and insulin-use status), and hovering over the icon reveals this description to the patient. Me indicates the patient's value, and hovering over the icon displays historical values. Literacy-level appropriate educational materials (hyperlinks) are paired with each measure. Message Your Doctor allows patients to send a secure message to members of their health care team. Online patient support community allows users to navigate directly to the American Diabetes Association (ADA) support community; a separate ADA account (username and password) is required. News Feeds provide newly published diabetes-related content, including recipes, discoveries, and new medications. FAQ provides answers to frequently asked questions regarding site features and navigation.

Participants randomized to the intervention arm are provided access to a version of MHAV embedded with MDC, as described in the *Introduction* section. Participants randomized to the intervention are advised to view MDC on a desktop or laptop device because the present version of MDC is not mobile friendly. Figure 2 shows a screenshot of MDC and illustrates its features [16,17]. Participants randomized to the usual care arm have access to the currently available version of MHAV, which includes the ability for patients to review pertinent health data, review medical information about their conditions, and communicate with their health care team.

### Data Collection and Measures

Enrolled participants receive 3 study questionnaires via REDCap at the associated time points (T_0_-T_2_): baseline questionnaire, 3-month follow-up questionnaire, and 6-month follow-up questionnaire. On the basis of pilot testing, we estimate the time to completion for the baseline questionnaire to be about 25 minutes and 20 minutes each for the 3-month and 6-month follow-up questionnaires. Participants are compensated US $40 for completing the enrollment questionnaire and US $35 each for completing the 3-month and 6-month follow-up questionnaires.

To describe the study population at baseline, we collect the following sociodemographic and clinical variables (Table 1). Health literacy is assessed by a validated 1-item screener asking respondents to rate their confidence independently filling out medical forms [18,19]. Consistent with previous studies, participants noting any lack of confidence are classified as having limited health literacy [20,21]. eHealth literacy is assessed by the 8-item eHealth Literacy Scale (eHEALS) [22]. The eHEALS uses a 5-point Likert scale ranging from *strongly disagree* to *strongly agree*. Total scores range from 8 (worst) to 40 (best). The presence of comorbidities (ie, hypertension and hyperlipidemia) is assessed by 2 clinicians who independently review patients’ problem lists and medications abstracted from the EHR, and disagreements are resolved by consensus.

Table 2 shows the primary and secondary outcomes and related measures contained within the study questionnaires and their associated time points. The same study measures are administered to all participants in both arms, except for the system use and user experience, which contain items unique to the participants’ assigned condition (ie, intervention vs control).

**Table 1:** Sociodemographic and clinical variables collected at baseline.

Variable and units or categories		Form of collection
**Age (years)**		Questionnaire
	<35	
	35-44	
	45-54	
	55-64	
	65-74	
	75-84	
	≥85	
**Ethnicity**		Questionnaire
	Hispanic or Latino	
	Non-Hispanic or Latino	
**Race**		Questionnaire
	White	
	Black or African American	
	American Indian or Alaska Native	
	Asian	
	Native Hawaiian or Other Pacific Islander	
	More than one race	
	Other	
**Gender**		Questionnaire
	Female	
	Male	
	Other	
**Marital status**		Questionnaire
	Never married	
	Married or partnered	
	Separated or divorced	
	Widowed	
**Education**		Questionnaire
	8 grades or less	
	Some high school	
	High school graduate or GED^a^	
	Some college or technical school	
	College graduate (bachelor’s degree)	
	Some graduate work or school	
	Graduate degree	
**Employment status**		Questionnaire
	Working full-time: 35 hours or more a week	
	Working part-time: less than 35 hours a week	
	Unemployed or laid off and looking for work	
	Unemployed and not looking for work	
	Homemaker	
	In school	
	Retired	
	Disabled: not able to work	
	Something else	
**Insurance**		Questionnaire
	An individual plan: the member pays for the plan premium	
	A group plan through an employer or union: the employer pays all or part of the plan premium	
	US governmental health plan (eg, Military, CHAMPUS^b^, Veterans Affairs, Medicaid, and Medicare)	
	I have not had an insurance plan in the past 12 months	
**Diabetes duration**		Questionnaire
	Years	
**Health literacy**		Questionnaire
	Adequate	
	Limited	
**eHealth literacy**		Questionnaire
	eHealth Literacy Scale score	
**Insulin status**		Questionnaire
	No	
	Yes	
**Previous diabetes self-management education**		Questionnaire
	No	
	Yes	
**Previous visit with dietician or nutritionist**		Questionnaire
	No	
	Yes	
**Comorbidities**		EHR^c^ abstraction
	Hyperlipidemia: no or yes	
	Hypertension: no or yes	
**Baseline clinical data**		EHR abstraction
	Glycated hemoglobin (%)	
	Systolic blood pressure (mm Hg)	
	Diastolic blood pressure (mm Hg)	
	Mean arterial pressure (mm Hg)	
	Low-density lipoprotein (mg/dL)	
	2019-2020 influenza vaccination status: no or yes	

^a^GED: Graduate Equivalency Degree.

^b^CHAMPUS: Civilian Health and Medical Program of the Uniformed Services.

^c^EHR: electronic health record.

**Table 2:** Outcome measures.

Outcomes		Measures	Variable type	Form of collection	Time points
**Primary** **outcome**					
	Patient activation	Patient Activation Measure-13 (R) [23]	Continuous	Questionnaire	T_0_^a^, T_1_^b^, and T_2_^c^
**Secondary cognitive** **and** **behavioral outcomes**					
	Diabetes self-efficacy	Perceived Diabetes Self-Management Scale [24]	Continuous	Questionnaire	T_0_, T_1_, and T_2_
	Diabetes knowledge	Short Diabetes Knowledge Instrument [25]	Continuous	Questionnaire	T_0_, T_1_, and T_2_
	Diabetes self-care	Summary of Diabetes Self-Care Activities [26]	Continuous	Questionnaire	T_0_, T_1_, and T_2_
	Diabetes medication adherence	Adherence to Refills and Medications Scale for Diabetes [27]	Continuous	Questionnaire	T_0_, T_1_, and T_2_
	Diabetes distress	Problem Areas in Diabetes Scale-5 [28]	Continuous	Questionnaire	T_0_, T_1_, and T_2_
	Understanding of diabetes health measures	Unique study-specific items to assess participants’ understanding of measures of diabetes health status	Categorical	Questionnaire	T_0_, T_1_, and T_2_
	Usability and satisfaction	System usability scale [29]	Continuous	Questionnaire	T_0_, T_1_, and T_2_
	System use data	Number of MHAV^d^ or MDC^e^ visits Duration of MHAV or MDC visits Number of MDC health data-related tasks performed (eg, view most recent low-density lipoprotein value) Number of MDC information-seeking tasks performed (eg, click links to embedded educational materials) Number of MHAV or MDC health management–related tasks performed (eg, utilization of embedded functionality to secure message health care team) Number of MDC social support seeking tasks performed (eg, click link to American Diabetes Association Online Community)	Continuous	System analytics (if available), self-report	T_2_
	User experience	Unique study-specific items to assess participants’ perspectives on specific features and functionality	Categorical and qualitative	Questionnaire	T_2_
	Clinical endpoints	Change in: Glycated hemoglobin Blood pressure Low-density lipoproteins Flu vaccination status	Continuous	EHR^f^ abstraction	T_0_, T_1_, and T_2_

^a^T_0_: baseline.

^b^T_1_: 3-month follow-up.

^c^T_2_: 6-month follow-up.

^d^MHAV: My Health at Vanderbilt.

^e^MDC: My Diabetes Care.

^f^EHR: electronic health record.

### Outcome Measures

#### Patient Activation

The primary outcome measure is the change in patient activation as assessed by the Patient Activation Measure (R) (PAM) [23]. The 13-item PAM (R) survey uses a 4-point Likert scale of response options ranging from strongly disagree to strongly agree and has excellent internal consistency reliability (Cronbach α=.87). The PAM-13 (R) survey item responses result in total raw scores ranging from 13 to 52, which are converted to the linear interval scale of patient activation scores, ranging from 0 (lowest activation) to 100 (highest activation).

#### Diabetes Self-Efficacy

The Perceived Diabetes Self-Management Scale (PDSMS) is used to measure diabetes self-efficacy (ie, how confident participants feel about their ability to perform multiple self-management tasks) [23]. The 8-item scale is scored on a 5-point Likert scale and has excellent internal consistency reliability (Cronbach α=.83). The total PDSMS score ranges from 8 to 40, with higher scores indicating a greater confidence in managing diabetes.

#### Diabetes Knowledge

The Short Diabetes Knowledge Instrument (SDKI) is used to measure diabetes knowledge, including diabetes diet, hypoglycemia symptoms, foot care, and the importance of physical activity [25]. The SDKI is a 13-item scale with scores ranging from 0 to 13, representing the number of items answered correctly, and has demonstrated good internal consistency reliability (Cronbach α=.73) in a diverse sample of older adults.

#### Diabetes Self-care

Change in diabetes self-care is measured using the Summary of Diabetes Self-Care Activities (SDSCA) [26]. The SDSCA is an 11-item questionnaire of diabetes self-management that assesses the following 6 aspects of the diabetes self-care regimen: general diet (2 items), specific diet (2 items), exercise (2 items), blood glucose testing (2 items), foot care (2 items), and smoking (1 item). Item responses use the metric *days per week*, except for a single item about smoking status, which is a *yes* or *no* item. Each of the 6 aspects is assigned a mean score based on the number of days per week.

#### Diabetes Medication Adherence

Change in diabetes medication adherence is measured using the Adherence to Refills and Medications Scale-Diabetes (ARMS-D) [27]. The 11-item ARMS-D scale has excellent internal consistency reliability (Cronbach α=.86). Responses range from 1=*none of the time* to 4=*all of the time* and are summed to generate an overall score ranging from 12 (best) to 48 (worst).

#### Diabetes Distress

The Problem Areas in Diabetes Scale (PAID-5) is used to measure changes in diabetes distress [28]. The 5-item unidimensional scale has scores ranging from 0 to 20, with higher scores suggesting greater diabetes-related emotional distress. The PAID-5 has excellent internal consistency reliability (Cronbach α=.86) and is associated with measures of depression.

#### Understanding of Diabetes Health Measures

Unique study-specific items are used to measure patients’ understanding of the diabetes health measures displayed within MDC. For example, patients are asked to identify the goal range for glycated hemoglobin (HbA_1c_), low-density lipoprotein (LDL) cholesterol, and systolic blood pressure.

#### Satisfaction With Usability

Usability of MDC is assessed by the 10-item System Usability Scale that measures users’ perceptions of ease of use, the likability of the interface, and overall satisfaction using a 5-point Likert scale (strongly disagree to strongly agree) [29]. The item scores are summed and then converted to a score ranging from 0 (worst) to 100 (best), with a score above 68 considered *above average* [30].

#### System Use Data

We are collecting MDC system use data, including the total number of visits, total duration, and use of embedded educational resources; secure messaging; participation in the online patient support community; and hovers over the information icon about diabetes health measures and diabetes news feeds.

#### User Experience

User experience is assessed by unique study-specific multiple-choice and open-ended questions that solicit participants’ perspectives on specific MDC features and functionality. For example, participants are asked to identify which features, if any, helped them better understand their diabetes health data and are asked to describe any problems they encountered using MDC.

#### Clinical End Points

Change in the following clinical endpoints is assessed by abstracting from the EHR the closest measurement on or before T_0_, T_1_, and T_2_ time points for each of the following measures: HbA_1c_, blood pressure, LDL cholesterol, and flu vaccination status. For the final time point (T_2_), we allow measures on or before T_2_ plus 2 weeks, as these measures can be reasonably assumed to reflect the study period.

### Data Analysis

#### Statistical Analysis Plan

The study is designed to evaluate the effects of MDC on patient activation (primary analysis) and explore the effects on other secondary cognitive and behavioral outcomes relative to the control group. We will use mixed-effects regression models to estimate potentially time-varying intervention effects while adjusting for the baseline measure of the outcome. Nonlinear associations will be modeled with regression splines. The mixed-effects model will use fixed effects for patient-level characteristics and random effects for health care provider variables, such as primary care physicians. We will provide point estimates with CIs for each follow-up and graphically depict our results. The analysis will follow a conservative intention-to-treat principle, and participants with missing values will be included along with those with complete data. Multiple imputation will be used to impute the missing values. The analysis with multiple imputation assumes missing at random (ie, the model properly handles missing data by including covariates associated with reasons for dropout). The characteristics of participants who do not complete the study or do not comply with the treatment will be compared for both conditions. Mixed-effect models will also be used to evaluate the effects of MDC on secondary outcomes. For dichotomous secondary outcomes, such as flu vaccination status, we will use mixed-effects logistic regression. Given the smaller effective size when modeling dichotomous outcomes, the model for the dichotomous outcomes will not support as many covariates as the model for continuous outcomes.

#### Primary Analysis

We will test the impact of MDC on patient activation compared with the control condition (Table 2). We hypothesize that participants assigned to MDC will experience greater improvements in patient activation than participants assigned to the control condition.

#### Secondary Analysis

In addition, we will test the effects of MDC on other behavioral and cognitive outcomes (Table 2). Finally, we will assess whether participants assigned to MDC experience greater improvements in HbA_1c_, blood pressure, LDL, and influenza vaccination status compared with those assigned to MHAV only.

#### Sample Size and Power

Assuming an up to a 20% dropout rate, approximately 240 patients (approximately 120 in each arm) are expected to complete the study. A conservative approach of a 2-sided *t* test performed at a 5% significance level would detect an effect size of 0.36 SDs for each continuous outcome with 80% power. In the context of the primary outcome PAM (R) survey and assuming a common SD of 12 points, this would be equivalent to detecting a true mean difference of 4.4 points; 4-point changes in the PAM (R) are associated with positive changes concerning particular diabetes self-care behaviors [31].

## Results

### Recruitment

**Figure 3:**
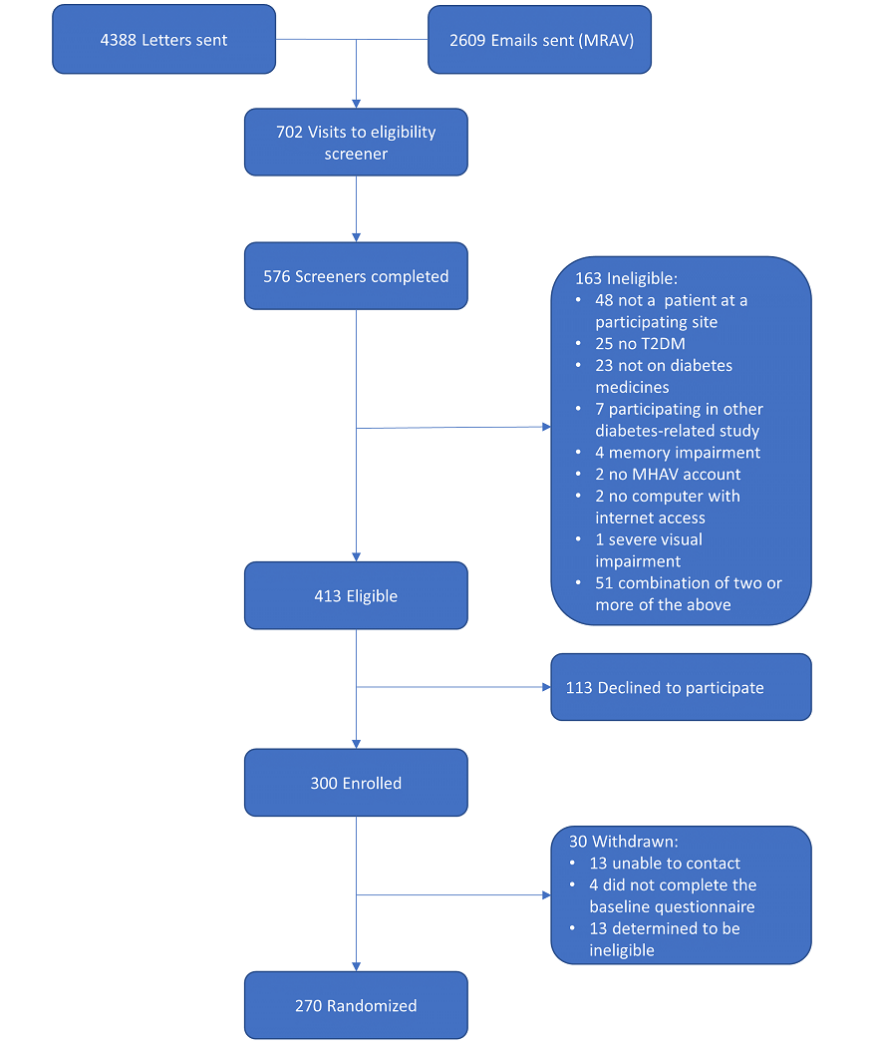
Recruitment and enrollment flowchart. MHAV: My Health At Vanderbilt; MRAV: My Research At Vanderbilt; T2DM: type 2 diabetes mellitus.

Figure 3 shows the flowchart of the recruitment process. Recruitment began in March 2020 and ended in May 2020. Throughout the recruitment period, 4388 unique letters were sent to patients identified as potentially eligible. Separately, 2609 unique emails were sent to patients who use MHAV and previously agreed to be contacted by email about research studies for which they might be eligible. As it was not possible for the study team to cross reference the list of those who were sent letters against the list of those who were sent emails, some overlap is possible. The letters and emails generated 702 visits to the web-based REDCap eligibility screener, resulting in 576 completed screeners. Of the 576 complete screeners, 163 (28.3%) were ineligible and 413 (71.7%) were eligible. Of the 413 eligible screeners, 113 (27.4%) declined to participate and 300 (72.6%) were enrolled. We administratively withdrew 10% (30/300) of those enrolled, and the remaining 270 participants were randomized.

### Participants

Of those randomized, most (214/267, 80.1%) were non-Hispanic White; 13.1% (35/267) were non-Hispanic Black; and 6.7% (18/267) reported being of another race, including American Indian or Alaska Native, Asian, Native Hawaiian or other Pacific Islander, more than one race, Hispanic Black, and Hispanic White. In addition, 43.7% (118/270) reported being 65 years or older. Furthermore, 10.1% (27/268) reported educational attainment of a high school degree or less, 33.6% (90/268) had limited health literacy, and 39.6% (106/268) had only a US governmental health plan (eg, Military, Civilian Health and Medical Program of the Uniformed Services, Veterans Affairs, Medicaid, and Medicare). Approximately one-third (82/270, 30.4%) were taking insulin, the mean duration of diabetes was 12.5 (SD 8.6) years, and the mean HbA_1c_ level at baseline was 7.1 (SD 1.3). The overall clinical population with diabetes has a somewhat different demographic distribution: 66.49% (5317/7997) are non-Hispanic White, 21.83%% (1754/7997) are non-Hispanic Black, and 42.34% (3386/7997) are 65 years or older. The mean HbA_1c_ of the 7997 patients within the overall clinical population and a laboratory value in the past 12 months was 7.40. As of October 2020, we have at least 95.6% (258/270) completion among participants through the 3-month follow-up assessment.

## Discussion

### Principal Findings

Our study will be one of the very few RCTs to evaluate a patient-facing diabetes digital health intervention delivered via a patient portal. Although diabetes digital health interventions have great potential, their impact has been limited due to the difficulty in integrating the interventions into routine care [32]. By embedding MDC into Epic’s MyChart platform with more than 127 million patient health records [33], our intervention is directly integrated into patients’ health care systems and is highly scalable and sustainable. Unlike independent health apps, patient portals, by their very nature, are integrated into routine care and therefore offer greater potential for uptake and sustained use [7]. The challenge for health systems and investigators is how to make the most of patient portals to improve care.

We designed MDC to enhance and expand on existing aspects of patient portals, including access to personal health data and education, to better support diabetes self-management. Although patient portals offer easy access to personal health data, previous research suggests that complex data displays—showing many tests in small format on a single page without any indication of their clinical significance—make it difficult for patients to find and correctly interpret a particular test result [7]. MDC uses a simplified infographic to indicate normal, modestly abnormal, and more severely abnormal results and literacy-level appropriate materials to help patients better understand their diabetes health data. Thus, this research will inform how different data displays and user interface designs impact patients’ ability to understand their personal health data.

Studies of other technology-enabled diabetes self-management solutions suggest additional strategies that may benefit patients [32,34]. These include analysis of patient-generated health data and tailored education and feedback [32,34]. To maximize scalability and sustainability, functionality enabling these strategies is best built directly into the EHR vendor’s patient portal platform (eg, Epic’s My Chart). Solutions built external to the platform may be more challenging to integrate into routine practice and run the risk of quickly becoming out of date and requiring reprogramming when vendors release platform updates. For this reason, we did not include these strategies in our intervention. However, EHR vendor solutions are emerging and may allow us to incorporate these additional patient engagement strategies into future iterations of MDC [35].

We designed MDC to be usable by the greatest number of patients, including those with limited health literacy [12]. Limited health literacy is typically associated with worse outcomes among patients with diabetes and can be a barrier to patient portal use [36,37]. Previous research has shown that patients with limited health literacy struggle to use patient portals because of complex medical terminology and a lack of literacy-level appropriate health information [20,21]. Although patient portals have the potential to worsen health inequities by further advantaging well-educated patients with greater resources, if designed and implemented appropriately, patient portals also have the potential to lower health literacy demands by ensuring that patients are presented with the health information and resources in a format that is convenient and easy to navigate and understand [38].

Our study population has a somewhat smaller proportion of racial or ethnic minorities than the overall clinical population, suggesting that additional strategies may be needed to increase adoption among these groups. Digital navigators—trained staff or volunteers who assist patients in accessing and learning how to use technology to meet their needs—have been used to increase patient portal adoption among vulnerable populations [20,39]. Smartphone use is increasingly common across different socioeconomic and racial or ethnic backgrounds, and for patients that lack broadband home internet connections, smartphones may be their only way to access the internet [40]. Thus, developing interventions suitable for mobile platforms may reduce barriers to adoption. Since the initiation of this trial, we have begun the development of a mobile-friendly version MDC that we hope will further increase its utility and accessibility.

Finally, given that racial and ethnic minorities are disproportionately affected by T2DM, future studies of MDC and other technology-delivered diabetes self-care interventions should consider using oversampling techniques, as demonstrated by Nelson et al [41,42], to recruit study populations that closely represent the overall population of patients with T2DM. Doing so will help ensure that technology-delivered diabetes self-care interventions are effective in the populations with the greatest need and inform any revisions to those interventions and/or their implementation needed to address disparities.

### Limitations

This study has important limitations. It relies on self-reported measures of patient activation and several secondary outcomes that are subject to social desirability and recall bias. However, the chosen measures are validated, widely used, and accepted, offering the advantage of being brief, inexpensive, and unobtrusive compared with more objective measures. Our study is powered to examine the effects of MDC on patient activation; therefore, analyses examining the effects of other outcomes (eg, self-care behaviors and HbA_1c_) and comparing the effects among subgroups (eg, patients with limited health literacy or poorly controlled diabetes) may be very informative but may also be underpowered. We hope that this study will serve as a pilot for a larger definitive trial evaluating the effect of MDC on clinical endpoints. Should MDC prove effective at increasing patient activation, the 6-month trial duration will not allow us to determine if the effect is temporary or sustained. A longer trial of a year or more in duration is needed to examine sustained effects. MDC is currently available only in English. This was necessary to increase the feasibility of designing the intervention and successfully completing this initial trial. However, diabetes disproportionately affects Spanish-speaking groups, so translation into Spanish will be an important goal, if MDC should prove beneficial. Finally, although patient portal interventions offer the advantages of direct integration into routine care, scalability, and sustainability, they are subject to inequities in patient portal adoption [43] and may appeal to more activated patients [44]. However, research shows that patient portal adoption is increasing [10,45], and if designed appropriately, patient portals could reduce health disparities [38,46]. Moreover, recent research finds that patient portal users have similar levels of patient activation as nonusers, although portal users are more likely to have internet access and a higher level of education [47].

### Conclusions

We expect that this study will help determine the effectiveness of MDC in increasing patient activation among patients with diabetes. Beyond this primary objective, we will also be able to examine data on secondary cognitive, behavioral, and clinical outcomes and users’ perceptions of and satisfaction with the intervention. Our findings and evolving patient portal functionality will inform the continued development of the intervention to best meet users’ needs and a larger trial focused on the impact of MDC on clinical endpoints.

## Abbreviations

ARMS-D: Adherence to Refills and Medications Scale-Diabetes
eCCM: eHealth Enhanced Chronic Care Model
eHEALS: eHealth Literacy Scale
EHR: electronic health record
HbA_1c_: glycated hemoglobin
LDL: low-density lipoprotein
MDC: My Diabetes Care
MHAV: My Health at Vanderbilt
PAID-5: Problem Areas in Diabetes Scale
PAM (R): Patient Activation Measure (R)
PDSMS: Perceived Diabetes Self-Management Scale
RCT: randomized controlled trial
REDCap: Research Electronic Data Capture
SDKI: Short Diabetes Knowledge Instrument
SDSCA: Summary of Diabetes Self-Care Activities
T2DM: type 2 diabetes mellitus
VUMC: Vanderbilt University Medical Center
